# MOVING FROM ENDORSEMENT TO ACTION: ALIGNING AFU EFFORTS WITH UNIVERSITY STRATEGIC PLANS AND GOALS

**DOI:** 10.1093/geroni/igac059.1726

**Published:** 2022-12-20

**Authors:** Sarah Walsh, Andrea Zakrajsek, Cassandra Barragan, Stephanie P Wladkowski

**Affiliations:** Eastern Michigan University, Ypsilanti, Michigan, United States; Eastern Michigan University, Ypsilanti, Michigan, United States; Eastern Michigan University, Ypsilanti, Michigan, United States; Bowling Green State University, Bowling Green, Ohio, United States

## Abstract

The Age-Friendly University global initiative has the opportunity to unite nations around the world towards making higher education spaces inclusive of older adults. Once a university joins the AFU Global Network and endorses the 10 guiding principles, the real work of living up to the principles begins. Each institution has unique geographical, political, social, and financial characteristics that require tailored approaches to this work. At our regional comprehensive university, we completed three separate analyses (external, internal, future directions) as part of our Environmental Scan. The goal of the Environmental Scan was to understand the initial strengths, weaknesses, and opportunities to age-friendliness and inclusiveness for older learners within our campus community. We utilized a multi-pronged data collection approach, including individual stakeholder interviews, online surveys of older leaners, policy analysis, and internal and external audits. Many aspects of data collection for the environmental scan occurred concurrently with data analysis. Data from each of the eight data collection methods were used to generate key findings and recommendations and will be presented. Furthermore, we found that aligning our recommendations with the University’s marketing and enrollment efforts as well as the University’s goals relating to diversity, equity and inclusion allowed for greater administrative support for strategies to increase age-friendliness on campus. with implications for similar work at other institutions. The Age-Friendly University global initiative has the opportunity to better support the inclusion of older adults in higher education. Our Environmental Scan of age-friendliness, from inception to completion will apply to institutions across the globe.

